# Effect of benznidazole on cerebral microcirculation during acute *Trypanosoma cruzi* infection in mice

**DOI:** 10.1038/s41598-022-25056-x

**Published:** 2022-12-06

**Authors:** Beatriz Matheus Souza Gonzaga, Samuel Iwao Maia Horita, Daniela Gois Beghini, Fabiana Gomes, Líndice Mitie Nisimura, Isabele Barbieri dos Santos, Vanessa Estato, Tania Cremonini de Araújo-Jorge, Luciana Ribeiro Garzoni

**Affiliations:** 1 Laboratório de Inovações Em Terapias, Ensino E Bioprodutos - Instituto Oswaldo Cruz, Fundação Oswaldo Cruz, Rio de Janeiro, Rio de Janeiro Brazil; 2 Laboratório de Pesquisa Em Malária, Instituto Oswaldo Cruz, Fundação Oswaldo Cruz, Rio de Janeiro, Rio de Janeiro Brazil; 3 Laboratório de Pesquisa Em Apicomplexa, Instituto Carlos Chagas, Paraná, Brazil; 4 Biotério Do Pavilhão Carlos Chagas, Instituto Oswaldo Cruz, Rio de Janeiro, Rio de Janeiro Brazil; 5 Laboratório de Imunofarmacologia - Instituto Oswaldo Cruz, Fundação Oswaldo Cruz, Rio de Janeiro, Rio de Janeiro Brazil

**Keywords:** Neuro-vascular interactions, Cerebrovascular disorders, Parasite host response

## Abstract

Central nervous system alterations was described in Chagas disease in both human and experimental models, leading to meningoencephalitis, stroke and cognitive impairment. Recently, our group demonstrated that acute infection by *Trypanossoma cruzi* leads to cerebral microvasculophaty in mice with endothelial dysfunction, capillary rarefaction, increased rolling and leukocyte adhesion. Only benznidazole and nifurtimox are available for clinical treatment, they have an efficiency of 80% in the acute phase and less than 20% in chronic phase. However, the effect of these drugs on brain microcirculation has not yet been evaluated. We hypothesized that early treatment with benznidazole could protect brain microcirculation during acute experimental Chagas disease. Swiss Webster mice were inoculated with 10^4^ trypomastigotes forms of *T. cruzi*, and after 24 h they were treated with 50 or 100 mg/kg/day of benznidazole for 14 consecutive days. In untreated infected mice, we observed cerebral microvascular rarefaction, increase in leukocyte rolling and adhesion, reduced cerebral blood flow, and increased CD3+ and F4-80+ cells in brain tissue. Early treatment with benznidazole at 100 mg/kg/day and 50 mg/kg/day prevented the occurrence of the alterations mentioned. Here, we show that BZ is able to protect the microcirculation and reduced brain inflammation in acute experimental Chagas disease.

## Introduction

Chagas disease (CD) is a neglected disease, endemic in Latin America, which affects approximately 7 million people worldwide^1^. CD is caused by the protozoan *Trypanosoma cruzi* (*T. cruzi*)^2^. This parasite can be transmitted by hematophagous insects of *reduvidae* family through the deposition of excreta after blood feeding, blood transfusion, congenital infection or oral ingestion of food/beverage contaminated by insects^3,4^.

Clinically, CD is divided into acute and chronic phases. The acute phase is characterized by high parasitemia and intense inflammatory response. Patients may have nonspecific symptoms, such as mild fever, or be asymptomatic^4,5^. The main cerebral manifestation at this stage is meningoencephalitis, which occurs mainly in children and immunosuppressed patients. In some cases, it can lead to patient death^6^. In 1–2 months the chronic phase begins, usually with an asymptomatic indeterminate phase, in which patients do not present clinical manifestation. Approximately 30% of patients will present some manifestation. The presence of parasites in different sites, including cardiac, digestive, and brain tissues, generates a low intensity but persistent inflammatory response, leading to different clinical forms of CD in tissues^7,8^. In the chronic phase, the main cerebral manifestation observed is ischemic stroke^9^.

Neurological involvement of CD was first described by Carlos Chagas in 1911^10^ and it can be defined as the finding of (1) trypomastigote on direct examination of the cerebrospinal fluid (CSF), (2) amastigotes on histopathological analysis of brain tissue, or (3) trypomastigote on direct examination of blood associated with neurological manifestations and clinical response after specific treatment^11^.

Meningoencephalitis is characterized by the presence of *T. cruzi* nest, inflammatory nodules composed mainly by parasites, mononuclear and glial cells in the brain^6,12,13^. Studies showed in an experimental model of CD that C3H/HE mice infected by the Colombian strain developed meningoencephalitis only in the acute phase of the disease, with the presence of lymphocytes, mainly T CD8+ cells, in the brain tissue^14^.

Ischemic stroke is a serious neurological manifestation and may or may not be associated with cardioembolism^15–17^. Studies suggest that the immune response is involved in intravascular processes in stroke caused by arterial occlusion or ischemic stroke. This event induces the production of reactive oxygen species (ROS), which trigger the coagulation cascade leading to the activation of complement, platelets, and endothelial cells. Also, increase in adhesion molecules, IFN-γ, TNF-α and inducible nitric oxide (iNOS) can be noted. Therefore, this process promotes vessel contraction, increasing brain vulnerability to ischemia^18^. The effects of stroke depend on which part of the brain is injured and how severely it is affected; a very severe stroke can cause sudden death^19^. Furthermore, human studies suggest that the pathogenesis of stroke may be related to microvascular changes in the brain^19^.

Our group demonstrated that mice acutely infected with *T. cruzi* presented increased cerebral oxidative stress and microcirculatory changes, such as, endothelial dysfunction, increased leukocyte rolling and adhesion, vascular obstruction, and reduced functional capillary density^20^.

The current therapeutic option for CD includes the trypanocidal nitrofurans compounds: benznidazole and nifurtimox. These drugs are 80% effective in the acute phase of CD, but less than 20% in chronic phase of disease^21^. However, the effect of these drugs on brain microcirculation is unknown. On the other hand, these drugs have side effects that can make patients drop out of treatment^22^, which occurs mainly when there is no medical follow-up^23^. Thus, dosage reduction is encouraged, in an attempt to observe beneficial effects with reduction of side effects^24^.

In this work, we present the effects of benznidazole on the brain of mice during experimental acute CD, evaluating inflammation, functional microcirculation and cerebral blood flow (CBF). Our results show evidence that benznidazole prevents the brain damage caused by acute *T. cruzi* infection^25^.

## Results

### Effect of benznidazole in parasitemia, body weight and survival

**Figure 1:**
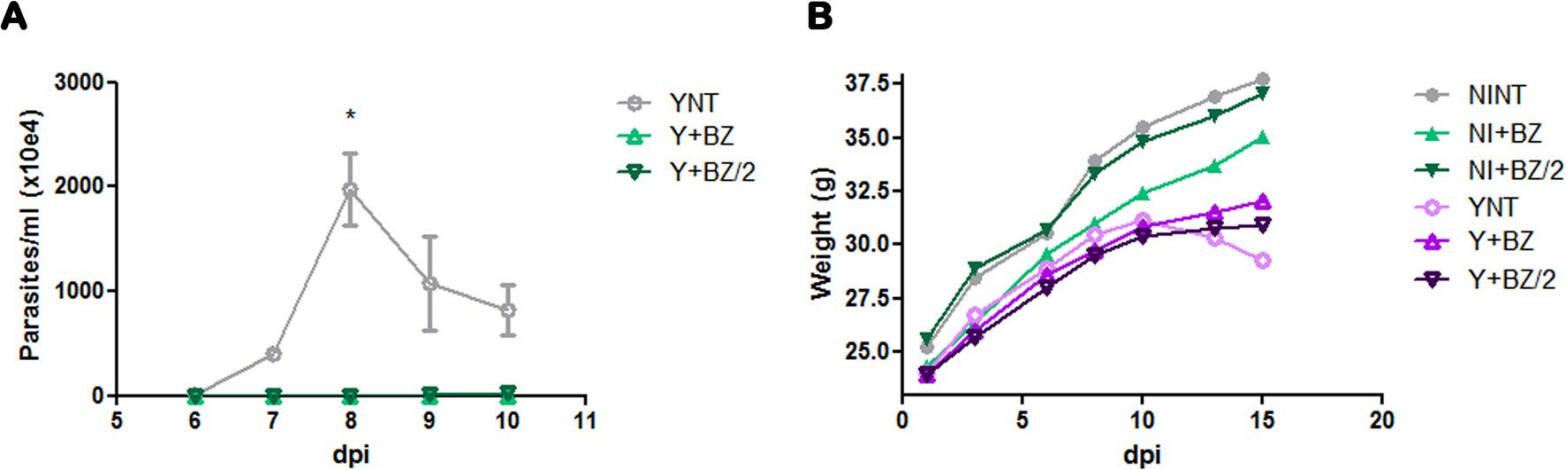
Characterization of the experimental model (Swiss Webster/Y strain). The following parameters were evaluated in a kinetic study: (**A**) parasitemia, (**B**) weight and (**C**) survivor. The parasitemia peak occurred at 8 dpi, and benznidazole treatment cleared parasitemia (**A**). T. cruzi infection induced body weight decrease in YNT group starting at 10 dpi, benznidazole treatment had no effect on this decrease (**B**). Benznidazole in both doses used impaired mortality (**C**). NI, non infected; Y, infected; NT, non treated; BZ, treated with benznidazole (100 mg); BZ/2, treated with benznidazole (50 mg); dpi, days post-infection.

After infection with *T. cruzi*, performed by intraperitoneal inoculum of 10^4^ bloodstream trypomastigote forms and treatment with BZ 24 h post-infection, we performed parasitemia at 6–10 dpi. The peak of parasitemia in non-treated animals (YNT group) occurred at 8 dpi with mean of 1970 × 10^4^ parasites/ml. Animals in Y + BZ group had no trypomastigotes forms in the blood at this time point and the Y + BZ/2 group presented very low parasitemia, with mean of 0.97 × 10^4^ parasites/ml (Fig. 1A). We evaluated mice’s body weight (Fig. 1B), and besides the tendency of reduction in body weight starting 10 dpi when comparing NINT control group to all infected group, no difference was observed in this parameter. NINT control group presented body weight of 37.75 ± 0.35 g, YNT group 29.30 ± 0.14 g; Y + BZ 32.05 ± 0.63 g and Y + BZ/2 group 31.8 ± 1.8 g. Treatment with both doses of BZ lead to impairment of the high mortality caused by the infection. At 22 dpi only three animals of YNT group out of fifteen lived.

### Benznidazole treatment prevents cerebral microvasculopathy caused by T. cruzi infection

**Figure 2:**
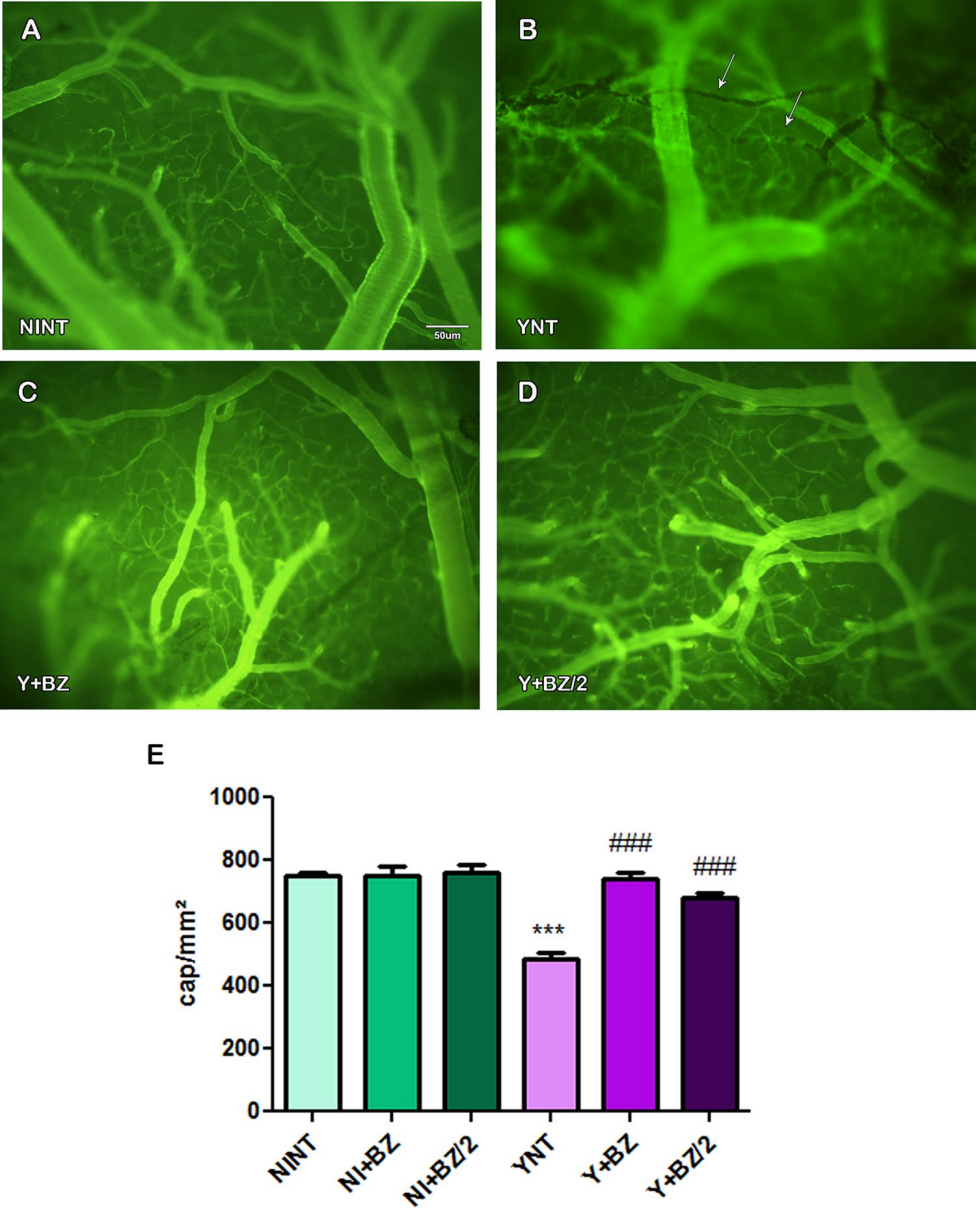
Capillaries functional density analysis by intravital microscopy in mice’s brain at 15 dpi. YNT group (**B**) had decreased number of capillaries in when compared to NINT group (**A**). Benznidazole treatment schemes prevented this decrease (**C**, **D**). Graphic indicate the number of spontaneously perfused capillaries (**E**). Arrows indicate non perfused capillaries. NT, non treated; BZ, treated with benznidazole (100 mg/kg/day); BZ/2, treated with benznidazole (50 mg/kg/day); dpi, days post-infection. *when compared to NINT; #when compared to YNT; *p < 0.05, **p < 0.01; ***p < 0.001.

We previously showed that acute *T. cruzi* infection causes cerebral microvasculopathy in mice (Nisimura et al.^21^) and in this study we decided to evaluate the effect of BZ treatment in preventing these microvascular changes. The intravital videomicroscopy confirmed our previous results, showing that non-treated *T. cruzi*-infected mice showed significant changes in microvascular perfusion when compared with NINT (Fig. 2A). At 15 dpi, we observed a reduction in number of brain perfused capillaries in YNT (484.75 ± 42.37 capillaries/mm^2^) compared with NINT (751.4 ± 23.57 capillaries/mm^2^—p < 0.001) (Fig. 2B). The abortive treatment with both the usual (BZ) and half dose (BZ/2) of benznidazole, initiated 24 h post-infection and maintained for 14 days, prevented these changes in microcirculation. The number of perfused capillaries in Y + BZ (742.5 ± 40.45 capillaries/mm^2^—p < 0.001) and Y + BZ/2 (682.16 ± 31.02 capillaries/mm^2^—p < 0.001) (Fig. 2C,D) was significantly higher when compared to the YNT group and was not distinct from NINT (Fig. 2E). The functional capillary density of non-infected animals treated with BZ or BZ/2 did not change in relation to the NINT control group.

### Benznidazole treatment prevents cerebral leukocyte-endothelium interactions during acute T. cruzi infection

**Figure 3:**
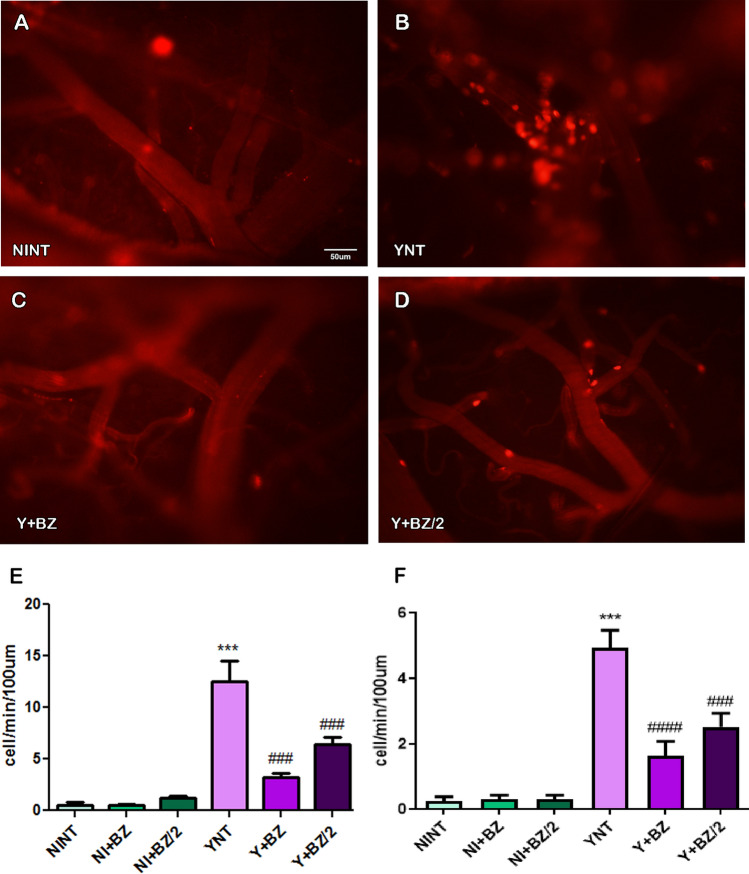
Analysis of leukocyte-endothelium interaction by intravital microscopy in mouse brain at 15 dpi. Increased number of leukocytes rolling and adhering to venules were observed in the YNT group (**B**) when compared to the NINT group (**A**). BZ treatment prevented both rolling and adhesion (**C**), and BZ/2 treatment only prevented the rolling of leukocytes (**D**). The graphic indicates the number of leukocytes rolling (**E**) and adherent (**F**) to venules. NI, nom infected; Y, infected; NT, non treated; BZ, treated with benznidazole (100 mg/kg/day); BZ/2, treated with benznidazole (50 mg/kg/day); dpi, days post-infection. *when compared to NINT; #when compares to YNT; *p < 0.05, **p < 0.01, ***p < 0.001, ****p < 0.0001.

Analysis of rhodamine-labeled leukocytes by intravital microscopy showed an increase in the number of leukocyte-endothelium interactions in the cerebral venular segment (Fig. 3E). The non-treated animals and infected with *T. cruzi* showed a high number of leukocytes rolling in the venules at 15 dpi (12.47 ± 4.08 cells/min) while Y + BZ and Y + BZ/2 groups exhibited 3.2 ± 0.99 cells/min (p < 0.001) and 6.4 ± 1.7 cells/min (p < 0.001) respectively, and NINT group presented 0.47 ± 0.61 cells/min. The number of adherent leukocytes in the YNT group (4.92 ± 1.21 cells/min) (Fig. 3B) were also higher then NINT control group (0.24 ± 0.32 cells/min—p < 0.001) (Fig. 3A) at 15 dpi. Abortive treatment with BZ and BZ/2 (Fig. 3C,D) impaired this increase (1.63 ± 1.10—p < 0.0001 and 2.51 ± 1.01, respectively) in infected mice (Fig. 3F). These results suggest that both proposed benznidazole treatment regimens, prevented leukocyte-endothelial interactions presented as the number of rolling and adherent leukocytes. The leukocyte rolling and adhesion of non-infected animals treated with BZ or BZ/2 did not change in comparison to the NINT control group.

### Benznidazole treatment prevents cerebral blood flow reduction induced by acute T. cruzi infection

**Figure 4:**
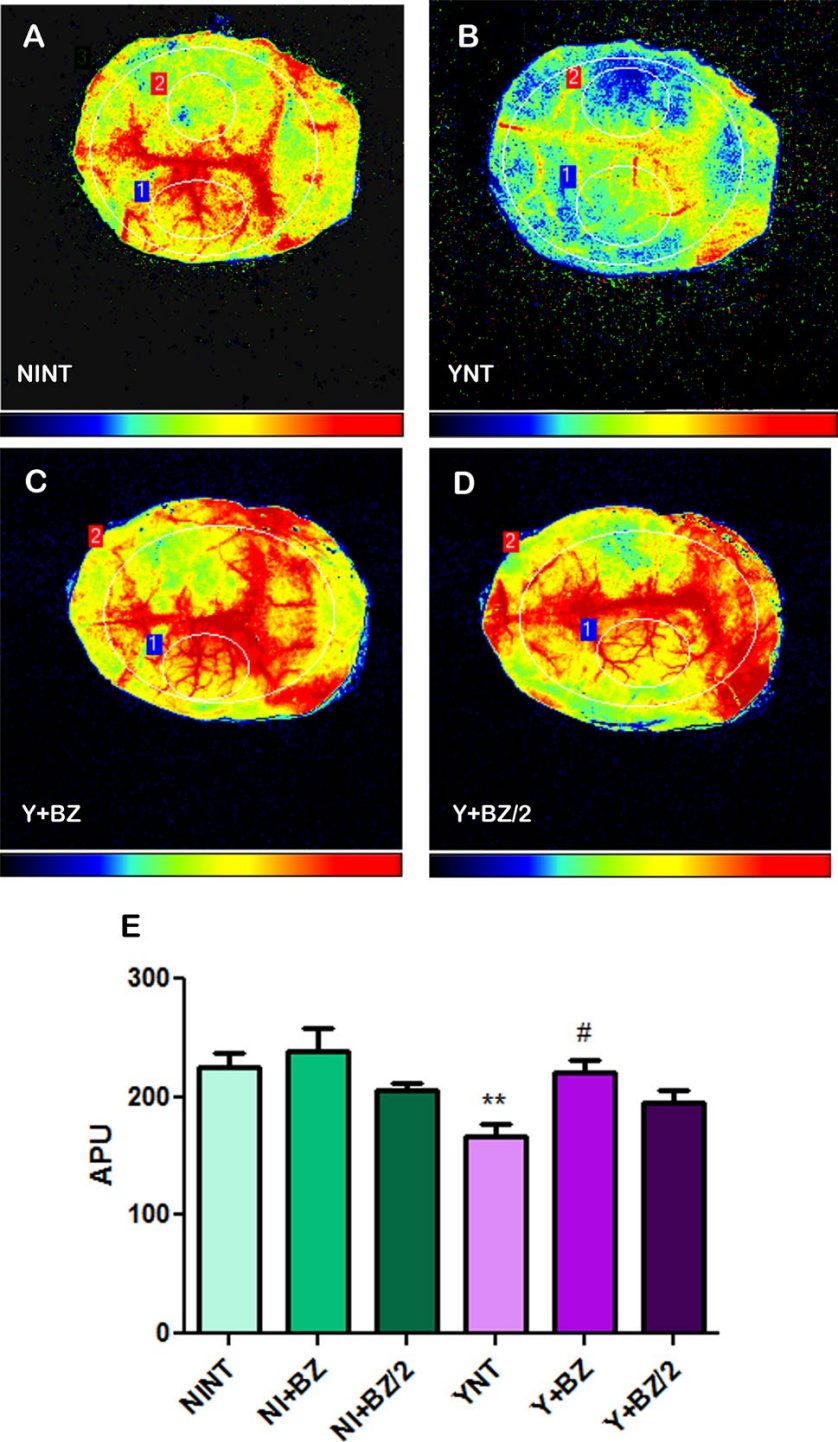
Analysis of cerebral blood flow by fluxometry at 15 dpi, showing decreased cerebral blood flow in the YNT group (**B**) when compared to NINT group (**A**). Treatments with BZ (**C** and **D**) prevented this decrease. Graphic indicates brain blood flow through arbitrary perfusion unit (APU) (**E**). NI, non infected; Y, infected; NT, non treated; BZ, treated with benznidazole (100 mg/kg/day); BZ/2, treated with benznidazole (50 mg/kg/day); dpi, days post-infection. *when compared to NINT; #when compared to YNT *p < 0.05, **p < 0.01, ***p < 0.001.

CBF was assessed using an LSCI system for the continuous, non-invasive measurement of cerebral vascular perfusion changes as measured in arbitrary perfusion units (APUs). The baseline values of CBF of non-infected animals were 224.94 ± 34.13 APUs (Fig. 4A). A decrease in CBF was observed in the YNT group (Fig. 4B) (167.05 ± 26.91 APUs—p < 0.01) at 15 dpi. This reduction was not observed in mice infected with *T. cruzi* treated with BZ (221.23 ± 25.70 APUs—p < 0.05) (Fig. 4C). The Y + BZ/2 group showed no changes in CBF when compared to the YNT group (195.19 ± 24.47 APUs) (Fig. 4D). This result showed that only benznidazole at its usual dose impaired the reduction of CBF caused by *T. cruzi* infection (Fig. 4E). The CBF of non-infected animals treated with BZ or BZ/2 did not change when compared to the NINT control group.

### Benznidazole treatment prevents the increase of F4/80+ e CD3+ cells in mice’s brain during acute T. cruzi infection

**Figure 5:**
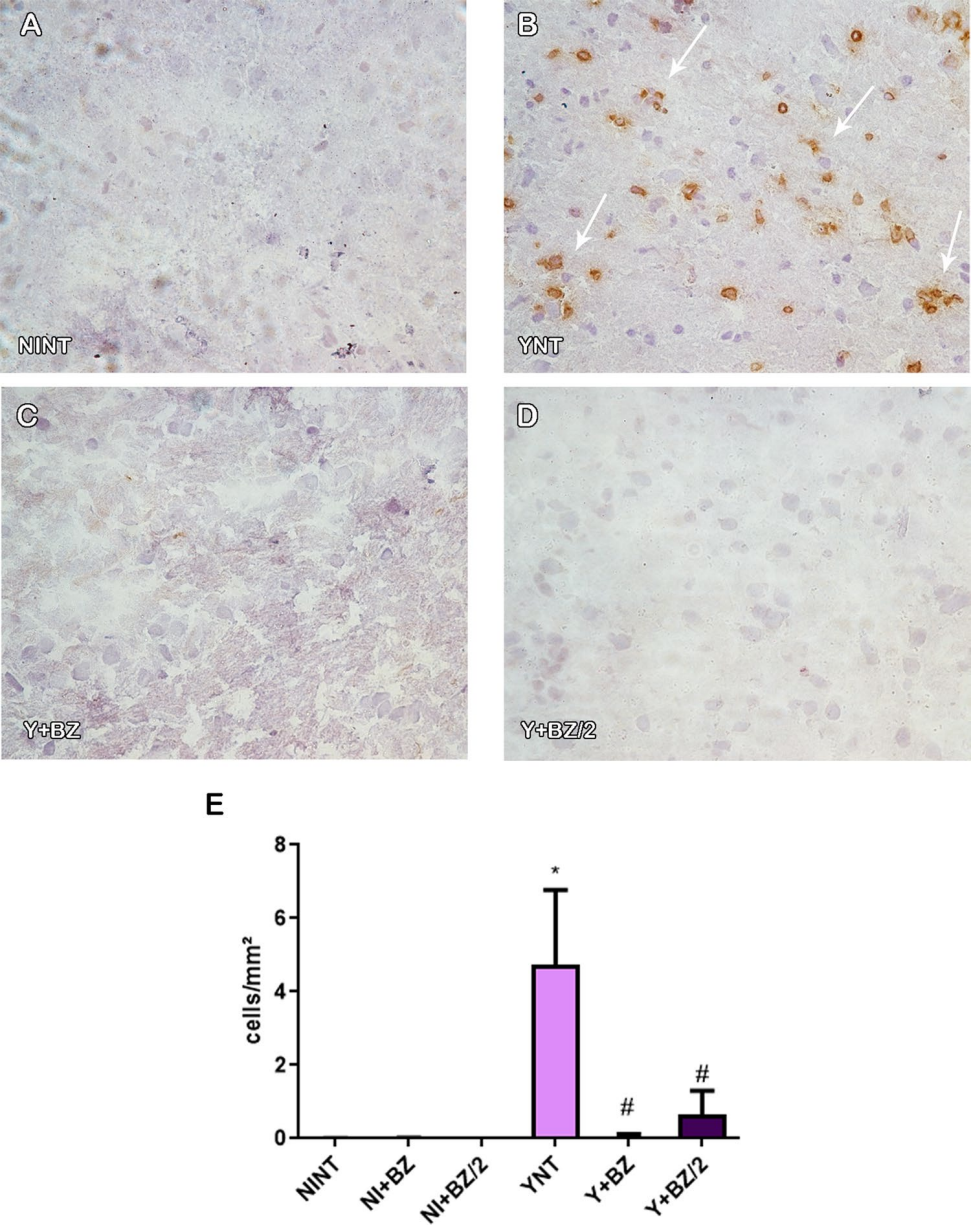
Analysis of the number of CD3+ cells by immunohistochemistry in mice’s brain at 15 dpi. YNT group (**B**) had an increased number of CD3+ cells when compared to NINT group (**A**). Treatment with BZ and BZ/2 (**C** and **D**) prevented this increase. Graphic indicate the number of cells per mm^2^ (**E**). Arrows shows positive cells. NI, non infected; Y, infected; NT, non treated; BZ, treated with benznidazole (100 mg/kg/day); BZ/2, treated with benznidazole (50 mg/kg/day); dpi, days post-infection. *when compared to NINT; # when compared to YNT *p < 0.05, **p < 0.01, ***p < 0.001.

Analysis of leukocytes subpopulation revealed that the number of CD3+ cells in the brain was increased in the YNT group (4.7 ± 4.5 cells/mm^2^) (Fig. 5B) at 15 dpi when compared to NINT control group (0 cells/mm^2^—p < 0.001) (Fig. 5A). Both abortive treatments with benznidazole prevented the increase of CD3+ cells in both concentrations used (BZ 0.07 ± 0.1 cells/mm^2^—p < 0.01; BZ/2 0.65 ± 1.45 cells/mm^2^—p < 0.01) (Fig. 5C–E).

**Figure 6:**
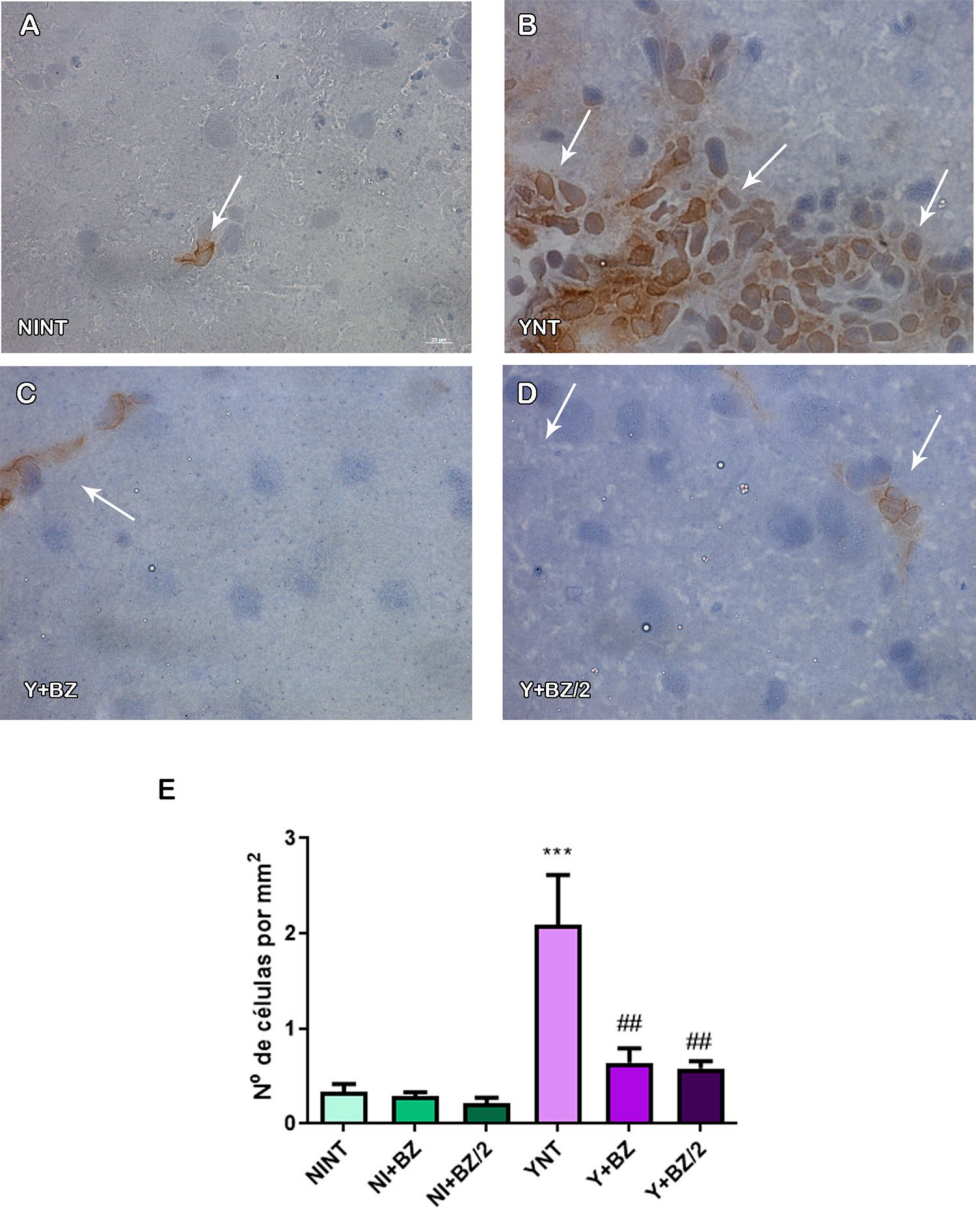
Analysis of the number of F4-80+ cells by immunohistochemistry in mice’s brain at 15 dpi. YNT group (**B**) presented increased number of F4-80+ cells when compared to NINT control group (**A**). Benznidazole treatment schemes (**C** and **D**) were able to prevent this increase. Graphic indicates the number of F4-80+ cells per mm^2^ (**E**). Arrows indicate positive cells. NI: non infected, Y: infected, NT: non treated, BZ: treated with benznidazole (100 mg/kg/day), BZ/2: treated with benznidazole (50 mg/kg/day), dpi: days post-infection. *When compared to NINT; # when compared to YNT *p < 0.05, **p < 0.01, ***p < 0.001.

F4/80+ cells were also analyzed and we observed that *T. cruzi* infection leads to an increase in the number these cells in YNT group at 15 dpi (2.09 ± 1.16 cells/mm^2^) (Fig. 6B) when compared to the NINT control group (0.33 ± 0.17 cells/mm^2^—p < 0,05) (Fig. 6A). Compared to the YNT group, this increase was prevented by benznidazole at the two concentrations used (BZ 0.64 ± 0.37 cells/mm^2^—p < 0.05; BZ/2 0.58 ± 0.16 cells/mm^2^, p < 0.05) (Fig. 6C–E). Non-infected animals treated with BZ or BZ/2 showed no change in the number of CD3+ and F4/80+ cells when compared to the NINT control group.

## Discussion

In the present study, we investigated the role of benznidazole at two different concentrations, on brain microcirculation and on inflammation in Swiss Webster mice during acute *T. cruzi* infection. We evaluated the number of spontaneous perfused capillaries, CBF, leukocyte-endothelium interactions (rolling and adhesion) and inflammatory infiltrate.

Animals were treated with the usual dose of benznidazole (100 mg/kg/day) or half of their usual dose (50 mg/kg/day). Early treatment with benznidazole started 1 day after infection and was maintained daily until 15 dpi, called abortive treatment.

The infection of Swiss mice with *T. cruzi* resulted in a peak parasitemia at 8 dpi and a 36% survival rate at 15 dpi, corroborating previous studies using the same experimental model^20,26^.

Our data confirmed that treatment with benznidazole at its usual dose of 100 mg/kg/day is able to eliminate parasitemia when using the early benznidazole treatment regimen (begin with 24 h after infection)^27,28^. Treatment with half the usual dose of benznidazole resulted in an expressive reduction of parasitemia. A study using the same animal model showed that treatment with 62.5 mg/kg/day of benznidazole starting at 7 dpi reduced parasitemia^29^. Furthermore, treatment with 50 mg/kg/day reduced parasitemia in an experimental model with Swiss Webster + Tulahuen strain^30^. Another study demonstrated that treatment with 30 mg/kg/day of benznidazole reduced parasitemia in C57BL/6 mice during late acute *T. cruzi* infection^31^.

Treatment with benznidazole in both concentrations leaded to an infected animal’s survival rate of 100% at 15 dpi.

We also verified a weight reduction in infected non-treated animals. However, this reduction was not altered by benznidazole treatment. Infected animals presented weight reduction between 10 and 15 dpi as previously described for the acute model using Swiss Webster mice infected with Y strain^32,33^.

Microvascular commitment was already verified in humans, and related to stroke development^17^. In the present study we demonstrated alteration on functional cerebral microcirculation in experimental acute CD through intravital microscopy, as described previously by our group^20^. Our analyses showed a decrease in the number of spontaneously perfused capillaries and increase in the number of leukocytes rolling and adherent in the brain of infected mice at 15 dpi. Benznidazole treatment at both doses used were able to prevent the reduction of the capillary density and prevented the increase on leukocyte adhesion and rolling. Treatment with 50 mg/kg/day of benznidazole in monotherapy presented tendency to impair the increase on leukocyte adhesion induced by the infection, however when 100 mg/kg//day of benznidazole were used, we observed a significant reduction in the number of adherent leukocytes.

As important as the results obtained on the effect of BZ on the microcirculation were the results with half the dose of BZ. When treating mice with half the dose of BZ we showed similar effect to the usual dose treatment scheme, being able to prevent functional capillary rarefaction and increase in leukocyte-endothelium interaction. These data suggest that BZ has a beneficial effect on brain microvascular changes even with dose reduction. Studies show that BZ can cross the blood–brain barrier, being distributed mainly to the brain, colon and heart^34,35^.

Another methodology used in our study were flowmeter which analyses CBF in real time thought an equipment of contrast imaging denominate laser speckle (LSCI). In this study we evaluated CBF thought LSCI in an experimental model of CD for the first time. We observed a reduction in CBF in animals infected with *T. cruzi* at 15 dpi*.* Nonetheless, treatment with 100 mg/kg/day or 50 mg/kg/day of benznidazole impaired the reduction of CBF caused by *T. cruzi* infection.

Strokes that occur during *T. cruzi* infection are mostly ischemic and could be associated with arrhythmias, congestive failure, apical aneurism and mural thrombus. However, strokes independent of cardiomyopathy where also described in literature^15,16^. In this context, a possible cause in these cases could be the commitment of the microcirculation, already described in humans with CD^9^, and the decreased blood flow.

Our results show intense alterations on functional cerebral microcirculation, with increase in leukocyte adhesion and rolling, decrease in the number of spontaneous perfused capillaries during acute CD, which could be contributing to the central nervous system alterations observed in chronic CD. Therefore, we intend to evaluate this alteration in chronic CD too.

Meningoencephalitis in acute CD is rare, but an important manifestation that occurs in children and immunosuppressed patients^6^. In humans is characterized by multifocal distribution of inflammatory cells and by the presence of inflammatory nodules in resolution^6,12^. As we observed the increase leukocyte-endothelial interactions, we aimed to investigate if the recruited leukocytes were indeed migrating to cerebral tissue. We evaluated the presence of CD3+ and F4/80+ cells in mice’s brain and verified that both subpopulations are present in large quantity on infected mice’s cerebral tissue. CD3+ cells were not found and a few F4/80+ cells could be observed in the cerebral tissue non-infected animals.

Our data demonstrate that early benznidazole treatment, with both 100 mg/kg/day and 50 mg/kg/day reduced the number of CD3+ and F4/80+ cells in the cerebral tissue of infected animals when compared to YNT group.

Study showed that immunocompetent C3H/He mice infected with Colombian strain of T. cruzi presented inflammatory infiltrate in the cerebral tissue mostly composed by T CD8+ cells and by macrophages and T CD4+ cells in small amount, during the acute phase of CD^14^.

Experimental model of acute CD, 62.5 mg/kg/day of benznidazole were able to induce the expansion of CD8+ splenocytes. This increase was larger when the total number of T lymphocytes in the spleen of infected animals were evaluated at 14 dpi^29^. However, our results demonstrate that at 15 dpi, that benznidazole in both concentrations used were able to decrease the number of total lymphocytes in the cerebral tissue. At Olivieri study, treatment was initiated at 7dpi, after high level of parasitemia, justifying the intense inflammatory response. In our treatment schemes, drugs were administrated 24 h after the infection, the trypanocide effect of benznidazole at the beginning of the infection was abortive, reducing drastically the number of parasites circulating. The inhibition the inflammatory response—reduction in the number of lymphocytes and macrophage in the cerebral tissue—was probably and result of this abortion of the infection.

In our study we elucidated some of the effects of the *T. cruzi* infection in the cerebral tissue in mice. In the acute phase of CD we can observe neurological commitment in children and immunosuppressed patients. Knowing what is happening in the brain during this phase of the infection could help us develop therapeutic strategies to act directly over this manifestation of the disease.

The pre-clinical data of the present study suggest that treatment with reduced BZ dose present similar effects to treatment with benznidazole usual dose, suggesting it could be used as a therapeutical alternative to prevent the development of cerebral microvascular alterations due to acute *T. cruzi* infection.

## Methods

### Ethics statement

All procedures were approved by the Oswaldo Cruz Foundation Animal Welfare Committee (License numbers LW-40/13 and LW-21/16) and were consistent with the USA National Institutes of Health Guide for the Care and Use of Laboratory Animals (NIH Publication No. 85-23, revised 1996). The study is reported in accordance with ARRIVE guidelines 2010.

### Animals

We used outbred male Swiss Webster mice (SWM) (age 6–8 weeks), weight 18–20 g) obtained from the Oswaldo Cruz Foundation Animal Facilities (CECAL, Rio de Janeiro, Brazil). Animals were housed for at least 1 week before parasitic infection under controlled conditions of light (12/12 h light–dark cycle) and temperature (22 ± 1 °C).

### Experimental groups

Mice were randomly divided into six groups. Non infected animals had 3 animals per group, and infected animals had five animals per group. Experimental groups were: non-infected non-treated (NINT), non-infected treated with benznidazole (NI + BZ), non-infected treated with half-dose of benznidazole (NI + BZ/2), *T. cruzi* (Y strain—TcII DTU)—infected non-treated (YNT), infected treated with benznidazole (Y + BZ) and treated with half-dose of benznidazole (Y + BZ/2). Infection was performed by intraperitoneal inoculum of 10^4^ bloodstream trypomastigote forms of *T. cruzi*. Age-matched, non-infected mice were maintained in identical conditions. For parasitemia, weight and survivor analysis three independent experiments were performed, for assessment of functional capillary density, leukocyte rolling and adhesion analysis and evaluation of CBF two experiments were performed. Total number of mice for parasitemia, weight and survivor were 9 for non-infected groups, and 15 for infected groups. For the other assessments 6 animals were analyzed per group.

### Drug and treatment scheme

Benznidazole (LAFEPE, BA, Brazil) was diluted in distillated water and administrated at the usual dose of 100 mg/kg and 50 mg/kg (half-dose). We used an early treatment regimen with benznidazole, which was initiated 1 day pos-infection and continued for 14 days. Treatment was administered in the morning by gavage. The animals were euthanized at 15 dpi after the procedures.

### Parasitemia, body weight and survivor

Parasitemia was individually assessed by the Pizzi-Brener method by direct microscopic counting of parasites in 5 ml of tail blood^25^. It was performed at different days post-infection (6–10 dpi). Body weight and survivor were regularly monitored for fifteen days post-infection (dpi).

### Intravital videomicroscopy in the brains of mice

The animals were anesthetized at 15 dpi by intraperitoneal injection with a mixture of xylazine (10 mg/kg) and ketamine hydrochloride (75 mg/kg). The animals were immobilized in a stereotaxic frame, and a cranial window was created by craniotomy with a high-speed drill to expose the cerebral microcirculation. The fluorescent dyes were intravenously injected into the tail. The animals were then placed on an upright fixed-stage of an intravital microscope with a mercury lamp (Olympus BX51/WI, USA) attached to a CCD digital video camera system. For Intravital videomicroscopy the assessment was blind. A student selected the animals through a code number, and gave to the machine operator. After the evaluation, the operator made the group analysis using the code number.

### Assessment of functional capillary density

After intravenous administration of 0.1 mL of 5% FITC-labeled dextran, microscopic images of the cerebral microcirculation were acquired by Archimed 3.7.0 software (Microvision, Evry, France) for online capillaries counting using Saisam software (Microvision, Evry, France). The functional capillary density, or the total number of spontaneously perfused capillaries (i.e., vessels with diameters less than 10 mm) per square mm of surface area (1 mm^2^).

### Leukocyte rolling and adhesion analysis

We labeled circulating leukocytes by injecting mice with intravenous rhodamine-6G (0.3 mg/kg). Fluorescent leukocytes were visible by epiillumination through the cranial window. We observed three randomly selected venular segments (30–100 mm in diameter and 100 mm lenght) for 60 s in each preparation. Leukocyte-endothelium interactions were evaluated by determining the number of (1) rolling leukocytes, defined as cells that cross the venular segment (100 mm) at a slower rate than circulating red blood cells (presented as the number of cells/min), and (2) leukocytes that adhered for at least 30 s to the venular wall.

### Analysis of cerebral blood flow

Cerebral blood flow was assessed using a laser speckle contrast imaging system (LSCI) (Perimed, Jarfalla, Sweden). LSCI provides a perfusion index proportional to the concentration and average velocity of red blood cells. The animals were anesthetized with a mixture of xylazine (10 mg/kg) and ketamine hydrochloride (75 mg/kg). After the craniotomy, the animals were placed under an LSCI light with wavelength of 785 nm. The distance between the laser light source and the skull was 10 cm as recommended by the manufacturer. To assess the cerebral blood flow in real time, a region of interest (ROI) was defined, covering almost the entire area available for imaging. Analysis of six laser speckle images per second and the relative cerebral blood flow of all animals were acquired using Perisof sofware (Perimed, Jarfalla, Sweden) and expressed as arbitrary perfusion units (APU). This assessment was blind, and the analysis was made using a code number.

### Immunohistochemistry

For immunohistochemistry the cryosections (5 μm) were fixed with ice-cold acetone (Merck) for 10 min. Non-specific binding was blocked using goat serum (Vector Laboratories) for 20 min. Primary antibodies, F4/80 BM8 and CD3 17A2 (Biolegend), were diluted in PBST and incubated overnight. For detection, ImmPRESS® HRP Goat Anti-Rat IgG Polymer Detection Kit was used with DAB (Dako) as a substrate. Images were obtained using Apotome (Zeiss) with camera for quantification.

### Statistical analysis

We expressed the results as the mean ± SEM for each group, and comparisons between groups were performed using unpaired t-tests or analysis of variance (ANOVA) followed by Bonferroni's multiple comparison test. Differences with p values of less than 0.05 were considered statistically significant. We used a commercially available, computer-based statistical package (GraphPad InStat 5.0, GraphPad Software Inc., La Jolla, CA, USA) for all calculations.

## Data Availability

The datasets used and/or analyzed during the current study are available from the corresponding author upon reasonable request.
